# Role of p38 MAPK Signalling in Testis Development and Male Fertility

**DOI:** 10.1155/2022/6891897

**Published:** 2022-08-31

**Authors:** Dandan Luo, Zhao He, Chunxiao Yu, Qingbo Guan

**Affiliations:** ^1^Department of Endocrinology, Shandong Provincial Hospital Affiliated to Shandong First Medical University, Jinan, Shandong 250021, China; ^2^Department of Endocrinology, Shandong Provincial Hospital, Shandong University, Jinan, Shandong 250021, China; ^3^Shandong Provincial Key Laboratory of Endocrinology and Lipid Metabolism, Institute of Endocrinology and Metabolism, Shandong Academy of Clinical Medicine, Jinan, Shandong 250021, China

## Abstract

The testis is an important male reproductive organ, which ensures reproductive function via the secretion of testosterone and the generation of spermatozoa. Testis development begins in the embryonic period, continues after birth, and generally reaches functional maturation at puberty. The stress-activated kinase, p38 mitogen-activated protein kinase (MAPK), regulates multiple cell processes including proliferation, differentiation, apoptosis, and cellular stress responses. p38 MAPK signalling plays a crucial role in testis development by regulating spermatogenesis, the fate determination of pre-Sertoli, and primordial germ cells during embryogenesis, the proliferation of testicular cells in the postnatal period, and the functions of mature Sertoli and Leydig cells. In addition, p38 MAPK signalling is involved in decreased male fertility when exposed to various harmful stimuli. This review will describe in detail the biological functions of p38 MAPK signalling in testis development and male reproduction, together with its pathological role in male infertility.

## 1. Introduction

The p38 mitogen-activated protein kinase (p38 MAPK) belongs to the family of MAPKs, which are involved in a variety of cellular processes including cell proliferation, differentiation, apoptosis, and cellular stress responses [1]. There are four isoforms of the p38 MAPK family, which are encoded by distinct genes: *p38α* (*Mapk14*), *p38β* (*Mapk11*), *p38γ* (*Mapk12*), and *p38δ* (*Mapk13*) [2–4]. Each of the p38 MAPK isoforms have been identified in the testis [5]. Recent studies have indicated that p38 MAPK signalling has broad physiological and pathological effects on male reproduction.

The testis is the male reproductive organ responsible for the generation of testosterone and sperm [6]. Testis organogenesis begins in the embryo with the development of the bipotential gonad into a testis, as opposed to an ovary, in a process called sex determination. In the XY gonad, male sex determination and embryonic testis development are regulated by an assortment of complex and interconnected molecular regulatory networks [6]. A defect in any XY gonad cells during testis development causes embryonic gonadal reversal and results in an improper testis [7]. In previous decades, studies have indicated that p38 MAPK signalling plays a vital role in male sex determination and embryonic testis development.

Postnatal testis development is a complex process involving the proliferation and differentiation of testicular somatic cells (Leydig cells and Sertoli cells), which culminates in testis maturation at puberty [8]. In adults, Leydig cells located at the testicular interstitium are the primary source of testosterone. Testosterone is essential for the development of the male phenotype and for spermatogenesis, alongside having various systemic endocrine effects outside of the testis [9–11]. Sertoli cells, the only somatic constituent of the testicular seminiferous epithelium, provide physical and nutritional support, as well as an immune-protective environment for the germ cells [12, 13]. Recent studies have indicated that p38 MAPK signalling has broad physiological effects on the function of both Sertoli and Leydig cells.

Over several decades, there has been a significant decreasing trend in male fertility, including a decline in the concentration of testosterone and the quality of sperm. The etiology of male infertility is very diverse, including environmental factors, sedentary lifestyle, aging, and systemic or testicular diseases. Testicular oxidative stress is considered to be the central mechanism leading to male infertility caused by these factors. As a stress-activated protein, p38 MAPK has a substantial role in the pathogenesis of many diseases. Growing evidence indicates that p38 MAPK signalling is involved in the reduction of male fertility in hazardous situations.

In this review, we described the key biological effect of p38 MAPK signalling on embryonic testis development and adult male fertility, in addition to discussing its potential role in male infertility during oxidative stress.

## 2. p38 MAPK Signalling Is Required for Male Sex Determination and Testis Development

In the majority of mammals, testes and ovaries are derived from the common bipotential gonads. Male fate is determined by SRY (sex-determining region, Y gene), which triggers the testis developmental pathway [14, 15]. SRY activates SRY-box transcription factor 9 (SOX9), which triggers supporting cells to differentiate into male Sertoli cells rather than female pregranulosa cells [16]. Sertoli cells subsequently signals embryonic primordial germ cells (PGCs) to differentiate into male germ cells. It is currently accepted that p38 MAPK signalling has a crucial effect on the fate determination of both somatic cells and PGCs in the XY gonad.

### 2.1. p38 MAPK Signalling Is Indispensable for the Fate of Sertoli Cell during Male Sex Determination

In 2009, a gene mutation was identified in mice that caused gonadal sex reversal, resulting in ovarian development in an XY embryo. This genetic mutation for boygirl is an early stop codon which disrupts the autosomal gene encoding mitogen-activated protein kinase kinase kinase 4 (MAP3K4) [17]. This team subsequently revealed that haploinsufficiency of MAP3K4 gives rise to the sex reversal phenotype in mice [18]. p38 MAPK is downstream of MAP3K4 in several cellular processes and is therefore inhibited in the testes of mice with a *Map3k4* mutation [19]. Mice with genetic ablation of both *p38α and p38β MAPK* cause XY gonadal sex reversal, which confirms that MAP3K4 regulates male sex determination via p38 MAPK signalling [20, 21].

**Figure 1:**
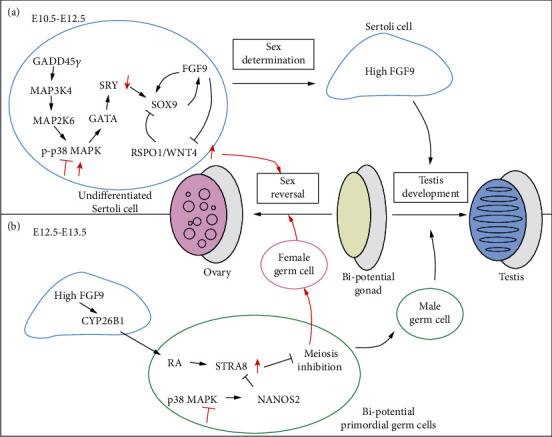
A summary of p38 MAPK signalling in sex determination in mice. (a) A diagrammatic representation of the sex fate decision of Sertoli cells. At around 10.5 dpc, p38 MAPK is activated by the GADD45*γ*/MAP3K4/MAP2K6 pathway. Then, p-p38 MAPK activates GATA4 by phosphorylation and subsequent expression of SRY. SRY upregulates SOX9 and FGF9 expression to induce differentiation into Sertoli cells (black arrows). At 12.5 dpc, testis cords have formed and morphological differences between the testis (blue) and the ovary (pink) are evident. Both inhibition and overactivation of p38 MAPK cause delayed onset and reduced expression of SRY, which permits WNT4 and RSPO1 to express in a female-specific manner, inducing ovarian development (red arrows). (b) A schema for the sex fate decision of XY PGCs. In XY gonads, the SRY-SOX9-FGF9 pathway prevents XY PGCs from differentiating into female germ cells by blocking the network of RA/STRA8 through CYP26B1. p38 MAPK signalling is activated in PGCs of XY gonads around E11.5 dpc and induces the expression of Nanos2. Nanos2 inhibits the expression of STRA8 to block PGCs from entering meiosis and promotes a male differentiation in XY PGCs (black arrows). The inhibition of p38 MAPK permits the expression of STRA8 in PGCs (red arrows).

Recent data has determined that growth arrest and DNA damage inducible gamma (GADD45*γ*) is the upstream kinase of MAP3K4 in male sex determination, since mice with a GADD45*γ* defect display XY gonadal sex reversal via the MAP3K4/p38 MAPK signalling pathway [20, 21]. Additionally, mitogen-activated protein kinase kinase 6 (MAP2K6) is the upstream kinase of the p38 MAPK-mediated male sex determination [19, 22]. Therefore, GADD45*γ*/MAP3K4/MAP2K6/p38 MAPK signalling determines the male sex determination (Figure 1(a)).

p38 MAPK signalling triggers the expression of SRY, which controls male sex determination. In XY gonads, a transient burst of SRY expression between 10.5 and 12.5 days postcoitum (dpc) in supporting cell progenitors initiates their commitment to a testicular fate (Sertoli cells) as opposed to ovarian development (pregranulosa cells) [23, 24]. In the absence of SRY, the gonadal primordium follows the alleged “default” pathway and develops into an ovary [15, 25]. p38 MAPK signalling ensures punctual expression of SRY via the transcription factor GATA-binding protein 4 (GATA4) in supporting cell progenitors, thereby prompting their commitment to a Sertoli cell fate [26]. Genetic ablation of p38 MAPK in an XY embryo causes XY gonadal sex reversal due to the delayed onset and reduced expression of SRY [20, 21].

Surprisingly, men with an overactivation of the p38 MAPK pathway caused by mutation in mitogen-activated protein kinase kinase kinase 1 (MAP3K1) display the male-to-female reversal phenotype [27]. MAP3K1 is one of upstream kinases of p38 MAPK. The mutation in MAP3K1 results in a gain of function and activates p38 MAPK. The overactivated p38 MAPK downregulates several male-specific genes, including SRY, SOX9, and anti-Müllerian hormone (AMH), and upregulates female-specific genes, including Wnt family member 4 (WNT4)/*β*-catenin and stimulated by retinoic acid 8 (STRA8), causing XY gonadal dysgenesis (Figure 1(b)) [28, 29].

Overall, both inhibition and overactivation of p38 MAPK result in male-to-female sex reversal. This reveals that the activity of p38 MAPK is vital for male sex determination. Minor changes in p38 MAPK activity can tilt the balance from testis-determining to ovary-promoting signalling. Additional experiments are therefore required to clarify the underlying mechanisms.

### 2.2. p38 MAPK Signalling Is Necessary for XY Germ Cell Fate Determination in Foetal Testis Development

Mammalian spermatozoa and oocytes are derived from the common embryonic PGCs, which colonise the nascent gonad, and later undergo sex-specific fate determination [30]. In foetal ovaries, the presence of retinoic acid (RA) and STRA8 signals PGCs to enter meiosis and induces them to differentiate into female germ cells at 12.5 dpc [31–34]. In foetal testes, fibroblast growth factors 9 (FGF9) in Sertoli cells blocks the expression of RA/STRA8 signals by inducing the expression of cytochrome p450 family 26 subfamily b member 1 (CYP26B1), a gene encoding an enzyme which degrades RA [35, 36].

p38 MAPK signalling is required for the fate determination of XY PGCs in embryonic testis development by inhibiting RA/STRA8 signalling. p38 MAPK signalling is activated in PGCs of XY gonads from around 11.5 dpc and induces the expression of Nanos2 (Nanos C2HC-Type Zinc Finger 2) in mice [35, 36]. Nanos2 inhibits the upregulation of STRA8 to block PGCs from entering meiosis and promotes male differentiation in XY PGCs [37]. Thus, inhibition of p38 MAPK permits the expression of STRA8 in PGCs [38]. However, it remains unclear whether the upstream kinase enzymes of p38 MAPK are involved in XY PGC fate determination.

As summarised in Figure 1(a), p38 MAPK is clearly necessary for determining the fate of pre-Sertoli cells and PGCs in XY gonads. Further research is needed in this area to clarify the underlying mechanisms at work.

## 3. The Role of p38 MAPK Signalling in Postnatal Testis Development

Testis development continues after birth. During this period, testicular somatic cells undergo proliferation and maturation, and spermatogenesis begins. p38 MAPK has a key role in proliferation, but not differentiation, of testicular cells during postnatal testis development.

Differential transcriptional profile analysis revealed that the signalling levels of the majority of MAPK genes are downregulated in mature and maturing Sertoli cells compared to immature Sertoli cells [39]. Perhaps p38 MAPK promotes Sertoli cell proliferation and blocks their maturation. Cecilia et al. discovered that inhibiting p38 MAPK attenuates the proliferation of cultured immature Sertoli cells [40]. The downregulation of p38 MAPK is required for Sertoli cell maturation. The overactivation of p38 MAPK caused by uninterrupted expression of tetraspanin-8 inhibits the maturation of Sertoli cells. The natural downregulation of tetraspanin-8 during puberty is considered a prerequisite for Sertoli cell maturation [41].

Research investigating the role of p38 MAPK in the proliferation and maturation of Leydig cells and the initiation of spermatogenesis is limited. However, the effect of another member of the MAPK family, extracellular-signal-regulated kinase (ERK), on postnatal testicular development has been well studied. ERK promotes immature Sertoli cell proliferation, suggesting p38 MAPK may have a similar role in postnatal testis development [42].

Regarding Leydig cells, ERK signalling is critical for maintaining its population in the adult testis. The Leydig cell-specific deletion of ERK1/2 results in Leydig cell hypoplasia, hypergonadotropic hypogonadism, and loss of fertility in adult mice [43]. Regarding germ cells, single-cell RNA sequencing data reveals that ERK1/2 signalling is activated in undifferentiated spermatogonia and begins to decrease during the spermatogonial stem cell- (SSC-) to-progenitor transition [44]. In addition, the results from mice with germ cell-specific deletion of ERK1/2 confirm that ERK1/2 signalling is predominantly activated in SSCs to maintain their undifferentiated state [45]. Therefore, ERK1/2 is necessary for SSC self-renewal and proliferation, but not for the initiation of spermatogenesis.

As a kinase essential for regulating cell proliferation, p38 MAPK appears to promote the proliferation but not the differentiation of Sertoli, Leydig, and germ cells in postnatal testis development. A testicular cell conditional p38 MAPK knockout mouse is required to prove this inference.

## 4. p38 MAPK Signalling Regulates Male Fertility

Male fertility depends upon spermatogenesis, which is the generation of mature spermatozoa. Spermatogenesis is tightly regulated by mature Sertoli and Leydig cells. Recent studies have indicated that p38 MAPK has a broad range of physiological roles in male fertility, including self-renewal and differentiation of SCCs and regulation of the functions of testicular somatic cells.

### 4.1. p38 MAPK Signalling Plays Multiple Roles in Various Biological Processes of Germ Cells

In adult testes, some SSCs maintain the stem cell pool through self-renewal, while others differentiate into spermatogonia to generate spermatozoa [46]. Spermatogonia proliferate and differentiate into primary spermatocytes, which undergo meiosis to produce haploid round spermatids [47]. These spermatids undergo metamorphosis into spermatozoa. Spermatozoa are subsequently released into the epididymis, where they undergo maturation to gain ability of motility [48]. p38 MAPK signalling has multiple roles in the biological processes of germ cells.

#### 4.1.1. Self-Renewal and Differentiation of SSCs

**Figure 2:**
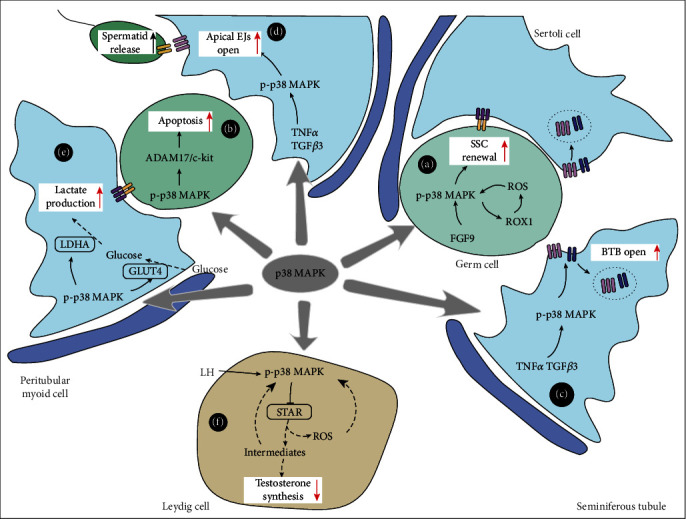
The molecular mechanisms of how p38 MAPK signalling regulates male fertility. p38 MAPK signalling is involved in (a) ROS-mediated SSC proliferation, (b) germ cell apoptosis, (c, d) dynamics of BTB and apical ESs, (e) glucose metabolism in Sertoli cells, and (f) negatively regulating testosterone synthesis in Leydig cells.

As displayed in Figure 2(a), p38 MAPK is involved in SSC self-renewal under the regulation of FGF9, which is vital for SSC self-renewal [49]. FGF9 activates p38 MAPK to promote the expression of ETS variant transcription factor 5 (ETV5) and B cell lymphoma 6 (BCL6), which are genes required for SSC self-renewal [50]. Inhibition of p38 MAPK inhibits FGF9-mediated SSC growth [51]. Similarly, an inhibitor of p38*α*/*β* MAPK (SB202190) prevents SSC self-renewal in mice *in vitro* [52].

A moderate concentration of reactive oxygen species (ROS) is required for SSC proliferation. ROS deprivation inhibits SSC proliferation by using ROS scavengers or ablating the gene NADPH oxidase 1 (NOX1), which is required for ROS generation. In SSC proliferation, p38 MAPK is activated when exposed to moderate ROS concentrations, while the inhibition of p38 MAPK prevents the expression of NOX1. These results suggest that NOX1/ROS/p38 MAPK is involved in ROS-mediated SSC proliferation (Figure 2(a)) [53].

#### 4.1.2. Spermatocyte Meiosis

DAZL (deleted in azoospermia-like) is a germ cell-specific RNA-binding protein, which has a vital role in spermatocyte meiosis [54, 55]. Recent studies suggest that p38 MAPK negatively regulates meiosis through DAZL. p38 MAPK activates MAPKAP kinase 2 (MK2), which phosphorylates DAZL and reduces the translation of DAZL-regulated target RNAs, resulting in a disorder in meiosis [56, 57].

#### 4.1.3. Apoptosis of Germ Cells

During spermatogenesis, germ cell apoptosis is a key event which controls sperm output by eliminating damaged or unwanted sperm. p38 MAPK induces germ cell apoptosis in a process mediated by several proteins (Figure 2(b)). p38 MAPK activates ADAM17 (A disintegrin and metalloprotease-17), a widely distributed extracellular metalloprotease. ADAM17 induces shedding of the extracellular domains of c-KIT, a glycosylated transmembrane protein before inhibiting cell survival signals [57–59]. p38 MAPK signalling is activated by downregulation of cold-inducible RNA-binding protein (CIRP), an RNA-binding protein expressed in normal testes and downregulated following heat stress, promoting germ cell apoptosis [53].

#### 4.1.4. Spermatozoan Maturation in the Epididymis

Spermatozoa in most mammalian species are kept completely motionless and viable for up to a few weeks in the cauda epididymis before ejaculation. Vigorous motility is initiated almost instantly upon sperm release from cauda during ejaculation. To gain fertilizing competence, they must go through a process called sperm capacitation [60]. In this process, sperm switch from progressive to hyperactivated motility and undergo a regulated release of acrosomal content in a process called the acrosome reaction (AR).

In both caudal and ejaculated spermatozoa, p-p38 MAPK is primarily localised to the upper midpiece of the spermatozoan tail where numerous mitochondria reside [61, 62], which suggests that p38 MAPK is closely related to sperm viability and motility. In caudal spermatozoa, p-p38 MAPK suppresses spermatozoan motility to ensure quiescence and survival via inhibiting mitochondrial respiratory capacity [63].

In ejaculated spermatozoa, the concentration of p-p38 MAPK is negatively associated with spermatozoan motility [64]. Consistent with this, p38 MAPK signalling inhibits both total and progressive spermatozoan motility [62]. Activated-p-p38 MAPK is involved in heat stress, and arachidonic acid (AA) caused the decline in sperm motility [65, 66]. In addition, p-p38 MAPK inhibits spermatozoan hyperactivated motility, which is a type of motility unique to capacitated spermatozoa [67]. Although p-p38 MAPK inhibits spermatozoan capacitation, it promotes the spermatozoan acrosome reaction [62].

### 4.2. p38 MAPK Signalling Regulates the Dynamics of the Blood-Testis Barrier (BTB) and Lactate Production in Sertoli Cells

The BTB consists of various types of junction connecting to adjacent Sertoli cells in proximity to the basement membrane, including tight junctions (TJs), adherens junctions (AJs), and gap junctions [68, 69]. The BTB separates the seminiferous epithelium into two distinct sections, the adluminal and basal compartments. Current researches indicates that p38 MAPK may regulate the dynamics of the BTB in a process mediated by transforming growth factor *β* (TGF-*β*) and tumor necrosis factor *α* (TNF-*α*) [70, 71]. TGF-*β* and TNF-*α* are crucial regulators of BTB dynamics [72].

As exhibited in Figure 2(c), p38 MAPK signalling is involved in the regulation of the BTB, mediated by TGF-*β*3. When TGF-*β*3 and its receptor T*β*R1 simultaneously bind to TAB1 and CD2AP, this complex activates p38 MAPK increases and subsequently disrupts the TJs by increasing the loss of TJ-associated proteins, such as occludin and zonula occludens-1, in cultured Sertoli cells [73]. This disruption is partially rescued by p38 MAPK inhibitor SB202190 [74]. Additionally, TGF-*β*3/p38 MAPK disturbs the dynamics of apical ectoplasmic specialisations (ESs), mediated by downregulation of the apical ES-associated proteins including cadherins and catenins (Figure 2(d)) [73]. The disassembly of apical ESs, the junctions restricted to Sertoli cells and spermatids, facilitates the release of spermatozoa [75, 76].

p38 MAPK is also involved in the dynamics of TJs and apical ESs, mediated by TNF-*α* [77, 78]. TNF-*α* transiently inhibits the steady-state protein concentrations of occludin, zonula occludens-1, and N-cadherin via activating p38 MAPK [79]. In addition, p38 MAPK promotes the transcription of a BTB protein, junctional adhesion molecule-B, in Sertoli cells under the regulation of IL-1*α* [80].

In addition, p38 MAPK in Sertoli cells serves as a significant regulator for glucose metabolism, making sure sufficient lactate supply for germ cells' energy substrate [81, 82]. Glucose deprivation activates p38 MAPK signalling in Sertoli cells, which subsequently increases the expression of glucose transporter type 1 (GLUT1), ensuring the uptake of glucose (Figure 2(e)) [83]. Activated p38 MAPK also promotes the expression and activity of lactate dehydrogenase (LDH) in Sertoli cells (Figure 2(e)) [84].

Generally, p38 MAPK is considered to be involved in the dynamics of the BTB and ESs to facilitate the migration of spermatocytes across the BTB and the release of spermatid. However, considerable *in vivo* research remains to be completed in this area.

### 4.3. p38 MAPK Is a Negative Regulator of Testosterone Synthesis in Leydig Cells

In Leydig cells, a series of steroidogenic enzymes are responsible for testosterone biosynthesis. The steroidogenic acute regulatory (StAR) protein regulates the rate-limiting step in steroidogenesis. Both LH/human chorionic gonadotropin (hCG) and cAMP enhance StAR expression [85]. Currently, p38 MAPK is considered a negative regulator of testosterone synthesis in Leydig cells (Figure 2(f)).

In Leydig cells and adrenal cell lines, which also produce testosterone, p38 MAPK inhibits the expression of StAR [86]. Overexpression of wild type, as opposed to the dominant negative form of p38 MAPK, significantly reduced the basal and cAMP-sensitive activity of the StAR promoter [87]. During steroidogenesis, mitochondria generate vast quantities of ROS. ROS and steroid precursors/metabolites lead to the activation of p38 MAPK. Activated-p38 MAPK inhibits the transcriptional activity of the cAMP-response element-binding protein (CREB) by phosphorylating CREB (p-CREB), which results in reduced expression of StAR via a feedback loop [88].

In males, serum testosterone levels decline with advancing age. This is referred to as age-related testosterone decline, and p38 MAPK is reportedly involved in this process [89, 90]. Elevated oxidative stress in aging Leydig cells suppresses steroidogenesis through the activation of the p38 MAPK/CREB/StAR pathway, a key mechanism behind age-related testosterone decline [91, 92]. Additionally, p38 MAPK upregulates the expression of cyclooxygenase-2 (COX2) in aging cells [93], which has an inhibitory effect on steroidogenesis in both young and old Leydig cells [92–95].

## 5. p38 MAPK Signalling Is Involved in Male Infertility Caused by Testicular Oxidative Stress Insult

**Figure 3:**
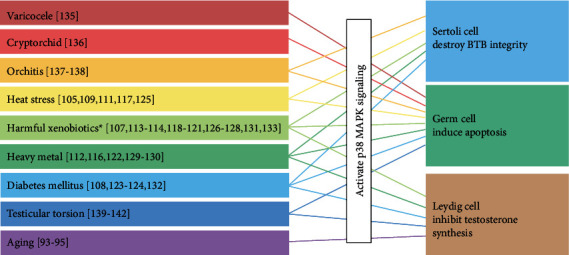
A summary of the stimuli which induce oxidative stress and activate the p38 MAPK pathway to injure testicular cells. ^∗^Harmful xenobiotics include PM2.5, organic compounds, pesticides, and plasticisers.

In recent decades, there has been a decline in the spermatozoan count and testosterone concentration worldwide [96–98]. These declines are linked to the effects of environmental contaminants, lifestyle factors, aging, and systemic and testicular diseases. Virtually, all of these factors cause overproduction of ROS, leading to oxidative stress in the testicular cells, which is the leading cause of male infertility [99–101]. As summarised in Figure 3, compelling evidence suggests that p38 MAPK, as a major regulator under oxidative stress, exacerbates the decline in male fertility.

### 5.1. Induce Germ Cell Apoptosis

Testicular cells, particularly germ cells, are highly sensitive to oxidative stress and undergo apoptosis in response to ROS. p38 MAPK participates in germ cell apoptosis via various pathways. Activated intrinsic apoptotic pathways, p38 MAPK/ROS/BAX/caspases, induce apoptosis of germ cells or ejaculated spermatozoan as a result of heat stress, hormonal stimulation, PM2.5, diabetes mellitus, and other stimuli [53, 78, 102–108]. Activation of BAX leads to the subsequent initiation of mitochondria-dependent death processes. Conversely, activated extrinsic apoptotic pathways triggered by p38 MAPK mediate germ cell apoptosis when exposed to selenium and lead [109, 110]. Additionally, germ cell apoptosis in response to bisphenol-A and nonylphenol is regulated by the activation of p38 MAPK/ADAM17/c-kit signalling [111].

However, contradictory results also exist. Activated-p38 MAPK alleviates heat stress-induced germ cell damage. p38 MAPK and its downstream substrate MAPKAP kinase 2 (MK2) phosphorylate HSPA1 of the HSP70 family, which renders germ cells more resistant to heat stress-induced apoptosis [112].

### 5.2. Disrupted BTB of Sertoli Cells

The BTB is the most significantly affected structure when Sertoli cells are exposed to oxidative stress from various harmful stimuli. Activated-p38 MAPK is involved in the disruption of the BTB integrity in multiple pathways. TGF-*β*3/p38 MAPK mediates the disruption of the BTB caused by physical/chemical factors, including PM 2.5, CdCl2, and heat stress [113–115]. p38 MAPK/Nrf2 is involved in BTB damage caused by PM 2.5 and polystyrene microplastics [116, 117]. Aroclor1254 disturbs the BTB by promoting endocytosis and degradation of junction proteins, including JAM-A, N-cadherin, and *β*-catenin, via the p38 MAPK pathway [118]. Glyphosate initiates calcium-mediated cell death in Sertoli cells by activating p38 MAPK [119]. ROS/p38 MAPK in the testes destroys the integrity of the BTB in diabetes mellitus patients, though the exact mechanism is unclear [120, 121].

### 5.3. Inhibited Testosterone Synthesis in Leydig Cells

p38 MAPK contributes to reduced testosterone concentrations in Leydig cells through the inhibition of expression or activity of enzymes related to testosterone synthesis or by inducing Leydig cell apoptosis [122]. Benzo (a) pyrene and beta-cypermethrin exposure prompted a ROS imbalance and activated p38 MAPK, which suppressed testosterone synthesis by preventing the expression of steroidogenic enzymes, such as cytochrome p450 family 11 subfamily a member 1 (CYP11A1), 3*β*-hydroxysteroid dehydrogenase (3*β*-HSD), and 17*β*-HSD [123, 124]. In Leydig cells, perfluorooctane sulfonate disrupts testosterone biosynthesis via the p38 MAPK/CREB/CRTC2/StAR signalling pathway [125]. Cadmium induces testosterone synthesis disorder via the TLR4/MAPK/NF-*κ*B signalling pathway [126]. p38 MAPK activation contributes to the Ni-induced testosterone synthesis disruption in rat Leydig cells [127]. Microcystin-LR (MC-LR) and elevated concentrations of glucose activate testicular macrophages, promoting their release of TNF-*α* [128, 129]. TNF-*α* binds to the TNF receptor 1 on the Leydig cells, thus activating the ROS/p38 MAPK signalling pathway, resulting in reduced serum testosterone levels. Both cordycepin and acrylamide induced apoptosis of Leydig cells by activating p38 MAPK signalling [130, 131].

In addition, ROS/p38 MAPK signalling has a role in male infertility in various testicular diseases, including varicocele [132], cryptorchidism [133], autoimmune orchitis [134, 135], and testicular torsion [136–139].

## 6. Concluding Remarks and Prospects for the Future

**Figure 4:**
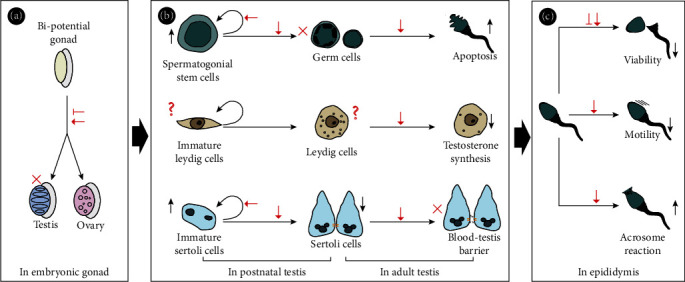
The roles of p38 MAPK in testis development and testicular function. (a) Both inhibition and overactivation of p38 MAPK disturb testis formation in embryonic gonad. (b) The effects of p38 MAPK on postnatal and adult testis: it maintains spermatogonial stem cell renewal and immature Sertoli cell proliferation; it inhibits spermatogenesis and Sertoli cell maturation; it reduces male fertility by promoting germ cell apoptosis, inhibiting testosterone synthesis, and disrupting integrity of the blood-testis barrier. (c) p38 MAPK regulates spermatozoan maturation, including maintaining spermatozoan viability, inhibiting spermatozoan motility, and improving spermatozoan capacitation in the epididymis. Red arrow indicates overactivation of p38 MAPK. Red line with end bar: inhibition of p38 MAPK. Shorter black arrow indicates upregulation or downregulation. Long black arrow: direction or flow. Red cross indicates a block. Red question mark indicates unknown.

The p38 MAPK signalling pathway plays important roles in testis formation and male fertility at the testis and epididymis levels (Figure 4). It is known that testis development and male fertility are regulated by the hypothalamus-hypophysis axis. Few studies revealed that p38 MAPK is involved in developmental migration and maturation of gonadotropin-releasing hormone (GnRH) neurons [140, 141]. In addition, p38 MAPK is expressed in kiss neuron and participants in the initiation of puberty [142, 143]. Further studies are needed to clarify the exact effect of p38 MAPK on the hypothalamus-hypophysis axis.

Furthermore, more rigorous studies based on gene-editing experiments are necessary to validate the present conclusion. Because the use of p38 MAPK inhibitors is a prerequisite for the generation of meaningful results, it may not be a true reflection of the testis *in vivo*, since the testis is a heterogeneous organ comprised of numerous cell types.

In addition, there is no doubt that p38 MAPK contributes to the reduced male fertility caused by various harmful stimuli such as environmental contaminants, systemic diseases, and aging. Several articles suggested that p38 MAPK signalling might be a potential effective therapeutical target. However, we do not think p38 MAPK per se is an ideal candidate, since p38 MAPK has both protective and detrimental effects in different testicular cells. The treatment based on the etiologies and ameliorating oxidative stress are more accessible, safe, and efficacious.

## Data Availability

The data supporting this systematic review are from previously reported studies and datasets, which have been cited.
